# In Silico Identification and Validation of Cuproptosis-Related LncRNA Signature as a Novel Prognostic Model and Immune Function Analysis in Colon Adenocarcinoma

**DOI:** 10.3390/curroncol29090517

**Published:** 2022-09-15

**Authors:** Yue Wang, Xulong Huang, Siyu Chen, Huajuan Jiang, Huanan Rao, Lijie Lu, Feiyan Wen, Jin Pei

**Affiliations:** State Key Laboratory of Southwestern Chinese Medicine Resources, School of Pharmacy, Chengdu University of Traditional Chinese Medicine, Chengdu 611137, China

**Keywords:** cuproptosis, colon adenocarcinoma, lncRNA, prognostic model

## Abstract

Background: Colon adenocarcinoma (COAD) is the most common subtype of colon cancer, and cuproptosis is a recently newly defined form of cell death that plays an important role in the development of several malignant cancers. However, studies of cuproptosis-related lncRNAs (CRLs) involved in regulating colon adenocarcinoma are limited. The purpose of this study is to develop a new prognostic CRLs signature of colon adenocarcinoma and explore its underlying biological mechanism. Methods: In this study, we downloaded RNA-seq profiles, clinical data and tumor mutational burden (TMB) data from the TCGA database, identified cuproptosis-associated lncRNAs using univariate Cox, lasso regression analysis and multivariate Cox analysis, and constructed a prognostic model with risk score based on these lncRNAs. COAD patients were divided into high- and low-risk subgroups based on the risk score. Cox regression was also used to test whether they were independent prognostic factors. The accuracy of this prognostic model was further validated by receiver operating characteristic curve (ROC), C-index and Nomogram. In addition, the lncRNA/miRNA/mRNA competing endogenous RNA (ceRNA) network and protein–protein interaction (PPI) network were constructed based on the weighted gene co-expression network analysis (WGCNA). Results: We constructed a prognostic model based on 15 cuproptosis-associated lncRNAs. The validation results showed that the risk score of the model (HR = 1.003, 95% CI = 1.001–1.004; *p* < 0.001) could serve as an independent prognostic factor with accurate and credible predictive power. The risk score had the highest AUC (0.793) among various factors such as risk score, stage, gender and age, also indicating that the model we constructed to predict patient survival was better than other clinical characteristics. Meanwhile, the possible biological mechanisms of colon adenocarcinoma were explored based on the lncRNA/miRNA/mRNA ceRNA network and PPI network constructed by WGCNA. Conclusion: The prognostic model based on 15 cuproptosis-related lncRNAs has accurate and reliable predictive power to effectively predict clinical outcomes in colon adenocarcinoma patients.

## 1. Introduction

Colon cancer is one of the most frequently diagnosed malignancies worldwide, with the fifth highest incidence and mortality rate of all cancers in 2020 [1]. Among them, colon adenocarcinoma is the most prevalent and aggressive subtype, accounting for approximately 80–90% of all colon cancer patients [2,3]. Additionally, the 5-year survival rate for patients with advanced stage is only 12.5%, compared to the higher survival rate for early-stage colon cancer [4]. Therefore, the development of a promising prognostic model is of great clinical value for the treatment of colon adenocarcinoma.

Copper is an important cofactor for the survival of prokaryotes and eukaryotes. Recent studies suggest that copper may play a role in the etiology and progression of cancer, as it was found that copper chelators significantly increased the number of natural killer cells and tumor-infiltrating CD8+ T, and slowed neuroblastoma growth [5]. It is worth mentioning that the level of copper was significantly different in the serum and tumor tissues of patients with lung, breast and thyroid cancers [6,7,8]. Because of its important role, copper may be a unique vulnerability in cancer progression [9]. Tsvetkov first introduced the concept of cuproptosis. Unlike known apoptosis, necroptosis, ferroptosis and pyroptosis, it relies on the accumulation of copper in the cell by binding directly to the lipoylated components of the tricarboxylic acid cycle, leading to the aggregation of lipoylated proteins and the loss of Fe-S cluster proteins, and ultimately to cell death through the stress response [10].

LncRNAs are defined as RNA transcripts greater than 200 nucleotides that do not encode proteins. They play a key role in various cellular processes in the cancer disease process [11,12,13]. Numerous studies have shown that lncRNAs can be involved in colon adenocarcinoma progression and immunotherapy through various pathways, such as m6A [14], ferroptosis [15], necroptosis [16], and autophagy [17]. In contrast, the value of the cuproptosis-related lncRNA prognostic model in colon adenocarcinoma has not received attention.

Therefore, we introduced colon adenocarcinoma-related data in TCGA, screened 15 cuproptosis-related lncRNAs by lasso regression, univariate Cox analysis and multivariate Cox analysis, and constructed a cuproptosis-related prognostic model based on these lncRNAs. The accuracy of the prediction model was further validated by various methods. In addition, immune-related functional analysis was investigated and potential drugs were screened for the treatment of COAD to provide a new reference for the prognosis of patients with colon adenocarcinoma.

## 2. Materials and Methods

### 2.1. Data Collection and Preparation

Colon adenocarcinoma (COAD)-related RNA sequence profiles, clinical data and tumor mutational burden data were downloaded from TCGA GDC (https://portal.gdc.cancer.gov/, accessed on 22 April 2022). RNA sequence profiles included 473 colon adenocarcinomas (COAD) and 41 adjacent normal healthy tissues. The Ensembl database (http://asia.ensembl.org/index.html, accessed on 23 April 2022) was used to distinguish between mRNAs and lncRNAs associated with these RNA sequence profiles.

### 2.2. Identification of Cuproptosis-Related lncRNAs

RNA sequence profiles were analyzed in combination with 10 cuproptosis-related genes identified by Tsvetkov et al. (FDX1, LIAS, LIPT1, DLD, DLAT, PDHA1, PDHB, MTF1, GLS, CDKN2A) to extract the expression files of cuproptosis-related genes [10] (Supplementary File S1). This file was further used with the lncRNAs of colon adenocarcinoma for co-expression analysis to obtain the expression file of cuproptosis-related lncRNAs (Supplementary File S2) and the filtering criteria (Pearson correlation coefficient > 0.4 and *p* < 0.001) [18].

### 2.3. Construction of Cuproptosis-Related Prognostic Signature

The expression files of cuproptosis-related lncRNAs and clinical data were combined and then univariate Cox analysis was performed with *p* < 0.05 (Supplementary File S3). Then, the lncRNAs obtained from the intersection of univariate Cox regression analysis and lasso regression were used in the multivariate Cox regression analysis [19]. The prognostic risk score for cuproptosis-related lncRNAs was calculated based on the linear combination of the regression coefficient (β) of the multivariate Cox regression model and its expression level [20]. The risk score was calculated by the following formula:

$$Risk\;score=\overset{N}{\underset{i=1}{\sum }}Coef_{i}\times x_{i}$$

where *Coef_i_* is the corresponding coefficient and *x_i_* is the expression level of lncRNA. Patients in the training and validation groups were divided into high/low-risk subgroups based on the median risk score. KM plotter survival curves were generated by using the R packages (survival and survminer) to compare overall survival (OS) between the high- and low-risk subgroups. Furthermore, to assess whether risk score could be regarded as an independent predictor of overall survival of COAD patients, univariate Cox and multivariate Cox regression analyses were performed with risk score, gender, age and metastasis status as variables using the R package (survival). The receiver operating characteristic curve (ROC) [21] was used to assess the predictive accuracy of the model, and all validations were performed simultaneously in the training and validation cohorts.

### 2.4. Construction of Nomogram

A nomogram is a graphical tool that is designed to approximate complicated calculation quickly [22]. It is a commonly used tool to estimate prognosis in oncology and medicine. Clinicians can look at the sum of all predictors for a given patient and predict the probability of survival at 1, 3 and 5 years. Nomogram was created by applying the R packages (survival, regplot, rms) and independent prognostic factors based on the downloaded COAD cohort, whose availability was assessed by the C-index [23].

### 2.5. Functional Enrichment Analysis and Immune-Related Functional Analysis

We explored differentially expressed genes in high- and low-risk groups using the R package (limma) with |log_2_FC| ≥ 1 and FDR < 0.05 as filters [24]. Then, the enrichment of differential genes was explored using Gene Ontology (GO) and the Kyoto Encyclopedia of Genes and Genomes (KEGG) with a filter condition of *p* < 0.05. Through GO analysis, we can obtain the cellular components (CCs), molecular function (MF) and biological processes (BPs) of differential genes. KEGG enrichment analysis was performed to observe in which pathways the differential genes were enriched. These processes are executed using the R packages ggplot2, enrichplot, circlize, org.Hs.eg.db, and clusterProfiler. Furthermore, to explore the immune infiltration in the COAD samples stratified by the signature, immune-related functional analysis was performed on expression data files and risk files using SSGSEA [25], and the results were visualized by heatmap visualization.

### 2.6. Tumor Mutational Burden Analysis and Sensitivity Assessment of Potential Drugs

Tumor mutational burden (TMB) data were downloaded from the TCGA database (https://portal.gdc.cancer.gov/, accessed on 22 April 2022). Additionally, the accuracy of the prognostic model was explored by TMB difference analysis and TMB survival analysis to see whether TMB affected the accuracy of the prognostic model. In addition, we used the R package (pRRophetic) to predict the IC50 of potential drugs in the high-risk and low-risk groups of COAD [26]. Additionally, the correlation between risk score and drug sensitivity was assessed by using a filter of *p* < 0.01 [27].

### 2.7. Construction of Competing Endogenous RNA (ceRNA) Networks and Protein–Protein Interaction Network (PPI) Based on Weighted Gene Co-Expression Network Analysis (WGCNA)

The lncRNAs and mRNA modules with the highest correlation coefficients were first screened by WGCNA. The lncRNAs in the MEblue module with the highest correlation coefficient were intersected with the differentially expressed lncRNAs in the TCGA data of colon adenocarcinoma, and then the miRcode database (http://www.mircode.org/, accessed on 25 August 2022) was used to predict the miRNAs interacting with these lncRNAs [28], and 28 pairs of interactions between 6 lncRNAs and 18 miRNAs were obtained. Databases such as miRNA (http://www.mirdb.org/miRDB/, accessed on 25 August 2022), miRTarBase (http://mirtarbase.mbc.nctu.edu.tw, accessed on 25 August 2022) and TargetScan (http://www.targetscan.org/, accessed 25 August 2022) were used to predict miRNA–mRNA relationships, and 325 mRNAs were identified. Cytoscape software (Version 3.8.0; Cytoscape Team, San Diego, CA, USA) was used to visualize the ceRNA network of lncRNA/miRNA/mRNA. Our obtained mRNAs were also used to construct PPI networks with STRING (https://string-db.org/, accessed on 25 August 2022) (high confidence level > 0.7 is considered significant) [29]. Meanwhile, Cytoscape’s MCODE plugin was used to extract the central genes from the PPI network. The potential signaling pathways of 325 target mRNAs were further probed by gene set enrichment analysis (GSEA) software (Version 4.2.3; Broad Institute, San Diego, CA, USA).

### 2.8. Statistical Analysis

All analyses were performed by Perl v5.30 (https://www.perl.org/, accessed on 19 April 2022) and R software v4.1 (https://www.r-project.org/, accessed on 19 April 2022). The differences between the two groups were assessed using *t*-tests (for normally distributed data) or Mann–Whitney tests (for non-normal distribution), and *p* < 0.05 was considered statistically significant. The relationships between risk score and drug sensitivity were determined by the Spearman’s correlation analysis [14]. *p* values were two-sided and all data with *p* < 0.05 were recognized as statistically significant.

**Figure 1:**
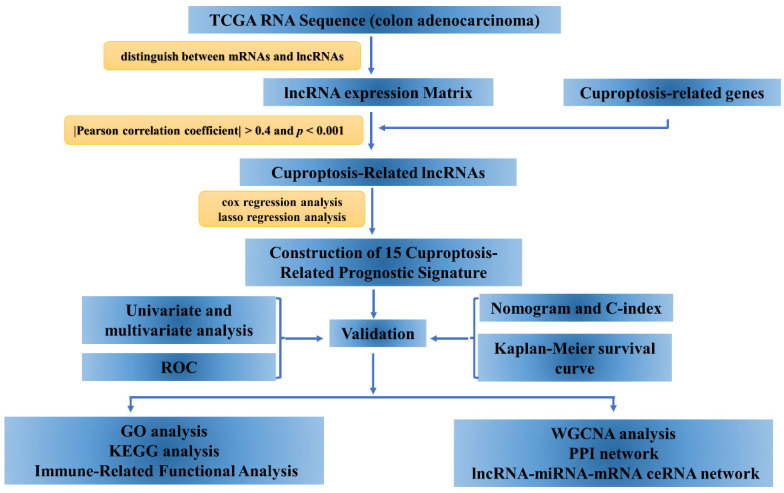
The flow chart diagram of the study.

The analytical procedure is shown in Figure 1.

## 3. Results

### 3.1. Derivation of lncRNA Prognostic Model

**Figure 2:**
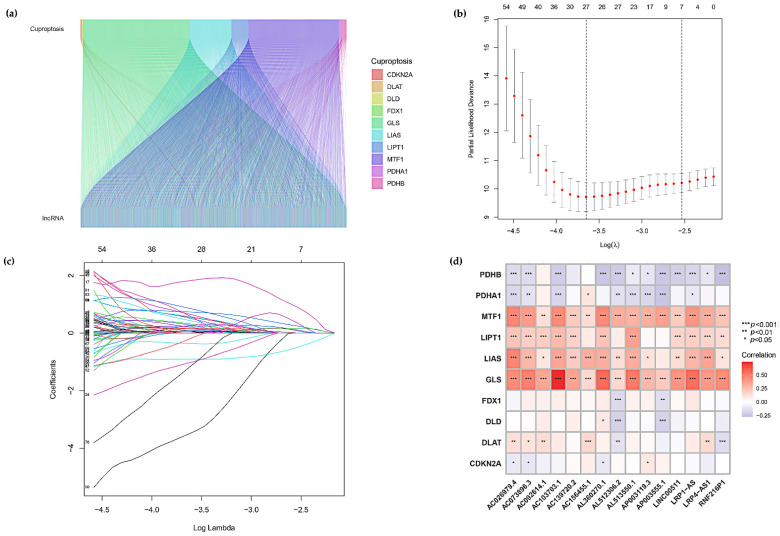
Screening for Prognostic lncRNA Signature. (**a**) Co-expression analysis of lncRNAs associated with cuproptosis-related genes in COAD. (**b**,**c**) The least absolute shrinkage and selection operator (LASSO) regression was performed with the minimum criteria. (**d**) Correlation analysis of 15 signature lncRNAs with 10 cuproptosis-related genes (horizontal coordinates are lncRNAs involved in model construction, blue represents negative correlation, red represents positive correlation).

The lncRNAs associated with cuproptosis-related genes were further extracted by co-expression analysis, and the co-expression results were further visualized (Figure 2a). A total of lncRNAs associated with CDKN2A, DLAT, DLD, FDX1, GLS, LIAS, LIPT1, MTF1, PDHA1 and PDHB were screened. lncRNAs were merged with the Colon adenocarcinoma survival data files in the TCGA database. Combining the survival data of the samples, we performed univariate Cox regression analysis to investigate the correlation between differentially expressed lncRNAs and OS of COAD patients. 99 lncRNAs that were strongly associated with OS (*p* < 0.05) were screened, and the best prognostic lncRNAs with significant eigenvalues were further validated and selected into high- and low-risk groups. 27 associated significant lncRNAs stood out in the lasso regression (Figure 2b,c), and based on the preliminary results of the lasso regression analysis screening we obtained 15 lncRNAs. The prediction model was constructed using multivariate Cox regression analysis (Table 1), containing AP003119.3, RNF216P1, AC156455.1, AL360270.1, AC073896.3, AC139720.2, AC092614.1, LINC00511, LRP4-AS1, AC026979.4, AC103703.1, AP003555.1, AL513550.1, LRP1-AS, AL512306.2. Among them, ten lncRNAs (AP003119.3, RNF216P1, AC156455.1, AL360270.1, AC139720.2, AC092614.1, LRP4-AS1, AP003555.1, AL513550.1 and AL512306.2) were harmful prognostic factors, and the others (AC073896.3, LINC00511, AC026979.4, AC103703.1 and LRP1-AS) were favorable prognostic factors. The correlation of the 10 cuproptosis-related genes with the 15 lncRNAs was analyzed by correlation heat map (Figure 2d), MTF1, LIPT1, LIAS, GLS showed positive correlations with most lncRNAs, and notably, MTF1, GLS showed positive correlations with all 15 lncRNAs (*** *p* < 0.001, ** *p* < 0.01, * *p* < 0.05).

### 3.2. Constructing a 15-lncRNA Prediction Signature of COAD

15 lncRNAs were identified by multivariate Cox regression analysis, and the prognostic risk score formula was obtained by constructing a model with the training group, and then the accuracy of the model was verified with the validation group. The risk score formula was constructed using the coefficients obtained from multivariate Cox regression, the risk score of each patient in the training group was calculated by the risk score formula, and the patients were classified according to the median value of the risk score into high-risk subgroup and low-risk subgroup, and similarly the patients in the validation group were divided into two subgroups of high and low risk. In the clinical data, a total of 446 COAD patients were selected after excluding samples with incomplete prognostic information. The clinical statistical analysis of the three groups (overall, training and validation) are shown in Table 2, the results showed that the *p* values for all clinical traits were greater than 0.05, indicating that there was no difference between the training and validation groups, proving that there was no bias in the sample grouping for the traits.

**Figure 3:**
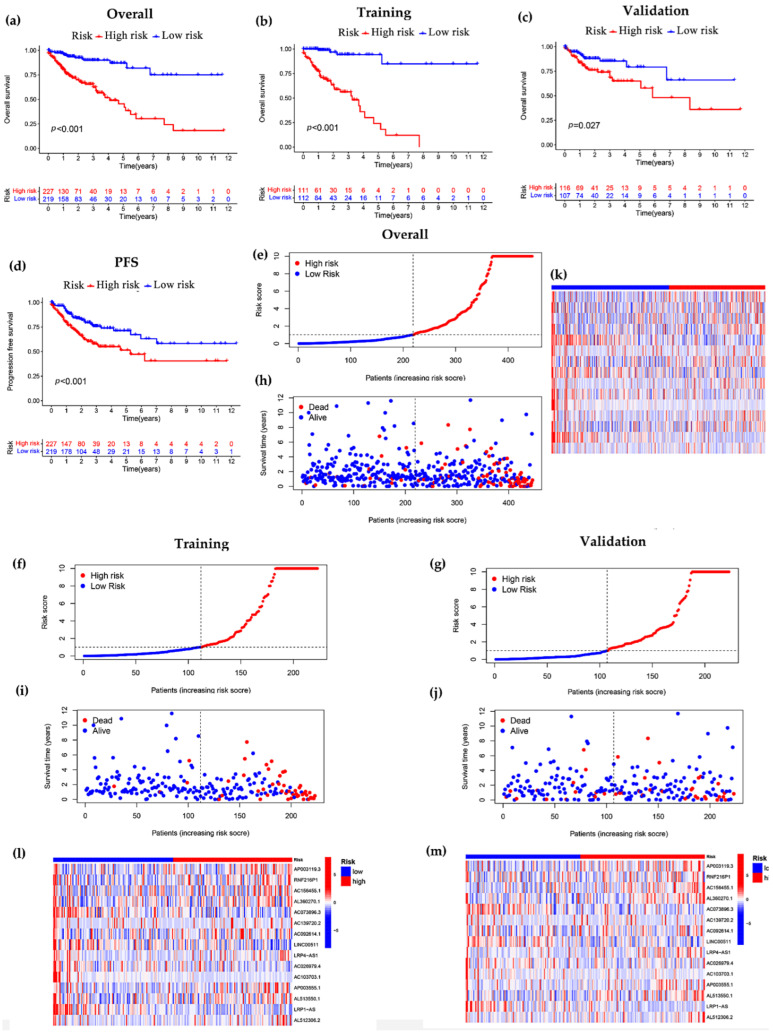
Construction of signature models for cuproptosis-related lncRNA in the overall group, training group and validation group. (**a**–**c**) Analysis of overall survival between high- and low-risk subgroups in overall, training and validation groups (compared to low-risk subgroup, high-risk subgroup showed significantly unfavorable prognosis, *p* < 0.05). (**d**) Exploration of differences in progression-free survival between high- and low-risk subgroups based on the overall subgroup (patients in the low-risk had significantly better PFS than high-risk patients, *p* < 0.001). (**e**–**g**) Risk score distribution (red is risk score of high-risk patients, blue is risk score of low-risk patients). (**h**–**j**) Relationship between risk score and survival time (horizontal coordinates indicate patients’ risk score, vertical coordinates indicate patients’ survival time. The number of patients who died increased with increasing risk score). (**k**–**m**) Heatmap of the expression of 15 lncRNAs in the high- and low-risk subgroups.

Further analysis revealed that the survival curves obtained from the overall, training and validation groups based on the prognostic risk model constructed with 15 lncRNAs showed that the overall survival (OS) was lower in the high-risk subgroup compared with the low-risk subgroup, and the difference between the two groups was statistically significant (*p* < 0.05, Figure 3a–c), which also indicated that our constructed model could distinguish patients in the high-risk and low-risk subgroups. Furthermore, progression-free survival (PFS) in the overall group revealed that PFS appeared lower in the high-risk subgroup compared with the low-risk subgroup, with a significant difference between them (*p* < 0.001, Figure 3d). The distribution of risk scores and survival status also showed that the higher risk scores were associated with more deaths among COAD patients in the overall, training and validation groups (Figure 3e–j). Meanwhile, we found that high expression of lncRNAs (AP003119.3, RNF216P1, AC156455.1, AL360270.1, AP003555.1, AL513550.1) was associated with increased risk in COAD patients, indicating that these lncRNAs are high-risk lncRNAs. Additionally, their high expression corresponds to shorter OS. In addition, with increasing patient risk, AC073896.3, LINC00511, AC026979.4, AC103703.1, LRP1-AS was down-regulated in expression, indicating that it is a low-risk lncRNA (Figure 3k–m).

### 3.3. Independent Prognostic Analysis of Predictive Model and Stratified Analysis of Clinicopathological Features

**Figure 4:**
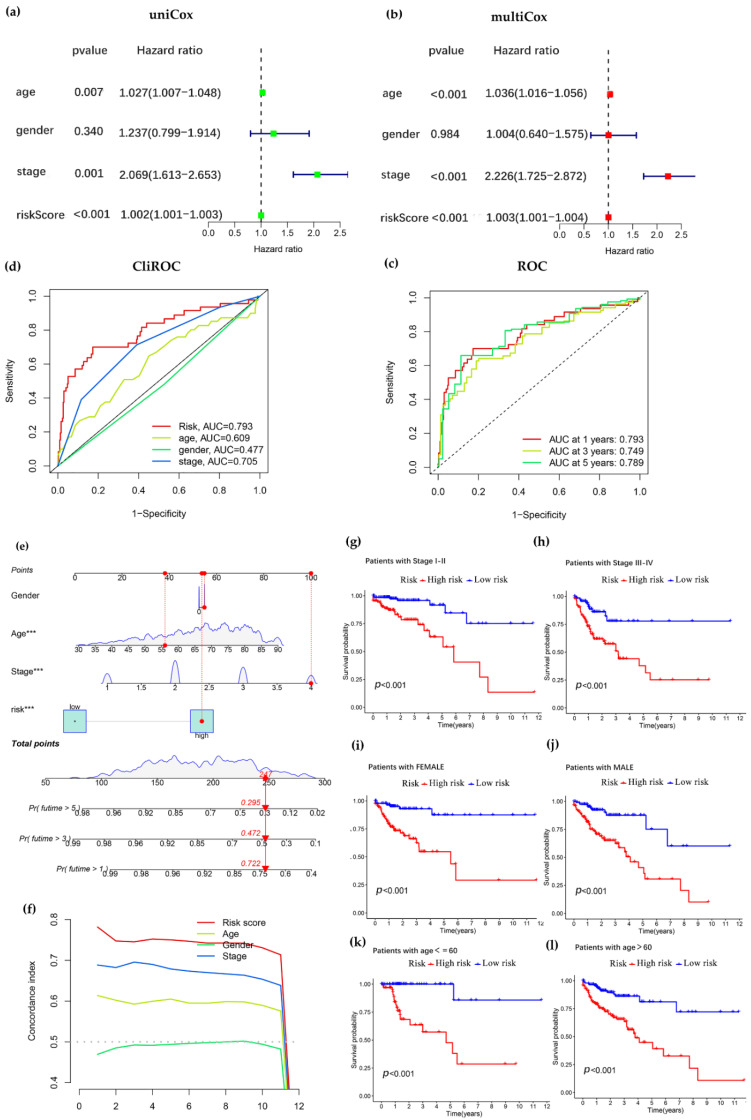
Validation of a prognostic model of cuproptosis-related lncRNA. (**a**,**b**) Univariate and multivariate analysis of clinic pathological factors of overall survival in COAD patients. (**c**) Comparison of receiver operating characteristic (ROC) curves for risk score models to predict 1-, 3-, and 5-year overall survival (OS). (The horizontal coordinate is the false-positive rate and the vertical coordinate represents the true-positive rate. The larger the area under the curve of the ROC curve, the higher the accuracy of the model’s prediction). (**d**) Comparison of ROC curves for risk score and other clinical factors (the risk score had the largest area under the curve, indicating that the constructed model was a better predictor of prognosis than other clinical characteristics in COAD patients). (**e**) Nomogram with risk score model and clinicopathological features (“***” indicates *p* < 0.001). (**f**) C-index curve combining risk score, stage, age and gender (The consistency index of the risk score was much greater than other clinical factors, and the risk score was more accurate in predicting survival in patients with COAD). (**g**,**h**) Different clinical stages of COAD were introduced for model validation (significant differences between high and low risk subgroups for stages I–II and III–IV of COAD, *p* < 0.001). (**i**,**j**) Kaplan–Meier survival curve with gender as an indicator (female vs. male). (**k**,**l**) Kaplan–Meier survival curve with age as an indicator (>60 years old vs. ≤60 years old).

To assess the independent prognostic ability of the constructed prognostic model, we performed univariate and multivariate Cox regression analyses on TCGA data, including age, gender, tumor stage and risk score, to verify whether the risk score of COAD-related lncRNA prognostic features could be used independently as an indicator of overall survival. The results of univariate Cox regression analysis showed that risk score (*p* < 0.001), stage (*p* < 0.001) and age (*p* < 0.007) were significantly associated with prognosis (Figure 4a). Multiple factors such as age, gender, stage and risk score were included in the multivariate Cox regression analysis, and risk score was found to be an independent predictor of prognosis in COAD patients (*p* < 0.001, Figure 4b). The above results suggest that the constructed model can be distinguished from the influence of other clinical features as an independent prognostic factor.

To determine whether the risk score model could be used as the best predictor of survival, age, gender, and stage were considered as candidate predictors. ROC curves were introduced to analyze the AUC for 1-, 3-, and 5-year prognosis, and it was found that the AUC was larger for 1 year (0.793), 3 years (0.749), and 5 years (0.789), indicating that the constructed model was more accurate in predicting patients (Figure 4c). Further, the ROC curves of the constructed model were plotted jointly with other traits, and it was found that risk score (0.793), and stage (0.705) had higher AUC among these factors, especially risk score had the largest AUC, indicating that our constructed model to predict patient survival was better than other clinical traits (Figure 4d).

To develop a clinically applicable method that can predict the survival probability of a patient, we used nomogram to construct a prediction model. Score is calculated by calculating the nomogram score based on the scores of each prognostic factor included in the nomogram to accurately predict the survival time of patients. We constructed an OS-based nomogram with gender, age, stage and calculated risk scores to accurately estimate the probability of patient survival at 1, 3, and 5 years, and the results showed a combined score of 446 for all clinical traits and survival rates of 0.722, 0.472 and 0.295 at 1, 3, and 5 years, respectively (Figure 4e). The criterion of *p* < 0.001 was met in risk score. In the C-index curves, the values were risk score, stage, age and gender in descending order, where risk score was more accurate and predicted the survival of COAD with greater accuracy (Figure 4f). To better assess the predictive power of the 15 cuproptosis -related lncRNA prognostic model, we performed a stratified analysis by stage, gender and age. The results showed that the *p* values of Kaplan–Meier survival curves for stage (stage I–II versus stage III–IV, Figure 4g,h), gender (female versus male, Figure 4i,j), age (≤60 years old versus >60 years old, Figure 4k,l) were less than 0.001, and the overall survival of the high-risk subgroup was significantly lower than that of the low-risk subgroup, indicating that our model is applicable to patients with early and advanced colon adenocarcinoma, as well as to patients of different genders and stages. These results suggest that the model constructed to predict patient survival is reliable and accurate.

### 3.4. Pathway Enrichment Analysis and Immune-Related Functional Analysis

**Figure 5:**
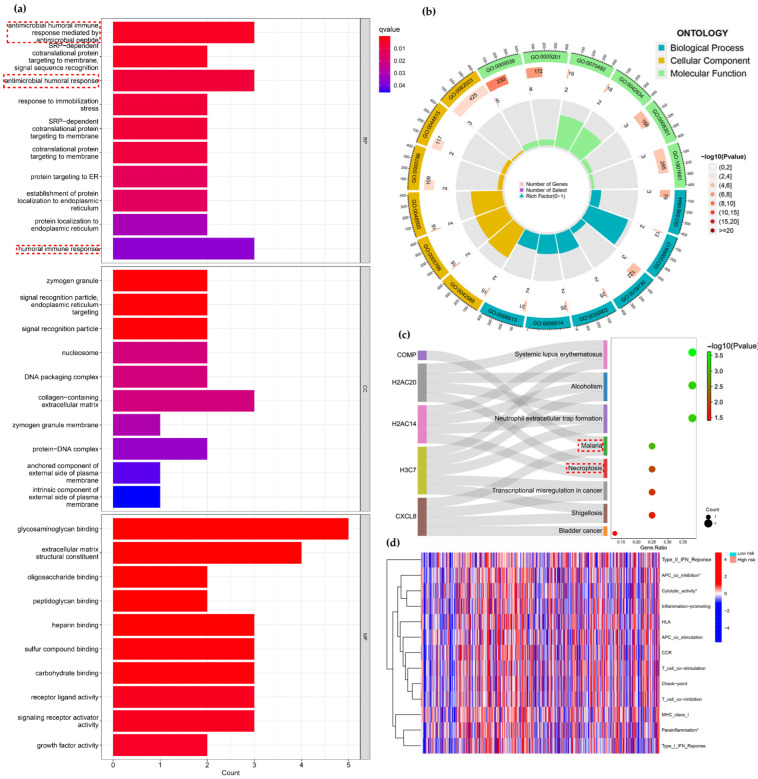
KEGG analysis and immune-related functional analysis. (**a**,**b**) GO enrichment analysis of risk differential genes. (**c**) KEGG analysis of risk differential genes. (**d**) Analysis of immune-related functions in high-risk and low-risk subgroups (* *p* < 0.05).

Through difference analysis, we identified differential risk genes between the high-risk and low-risk groups. The results of GO analysis mainly included biological process (BP), molecular function (MF) and cellular component (CC) (Figure 5a,b). The results of BP were enriched in humoral immune-related pathways and Signal recognition particle (SRP)-associated proteins. The results of Ahmadi et al. also suggest a strong relationship between colon adenocarcinoma and immunity [30]. CC processes focused nucleosome, DNA complexes and other processes that are closely related to tumor cell growth and proliferation [31,32]. MF enrichment in polysaccharide-binding components, activity of receptor ligands. Additionally, KEGG results showed that differential risk genes were associated with pathways such as malaria and Necroptosis (Figure 5c). Interestingly, some studies have shown a negative correlation between the incidence of malaria and mortality from colon and anal cancers in humans [33], which has been confirmed in several studies examining animal models. For example, the antitumor effect of a mouse model of lung cancer infected with malaria parasite is mediated by innate and adaptive immunity [34]. Necroptosis is a new form of programmed death that is closely associated with various cancers, including colon cancer [16]. The above findings suggest a potential relevance of these pathways to the mechanism of COAD.

Therefore, further analysis of immune-related functions was performed using ssGSEA (Figure 5d). We observed that there were significant differences between high-risk and low-risk groups in APC co inhibition (*p* < 0.05), Cytolytic activity (*p* < 0.05), Parainflammation (*p* < 0.05) among which APC co inhibition, Cytolytic activity and Parainflammation were actively expressed in low-risk group. The above results suggest that significant differences in prognosis between high- and low-risk subgroups may be related to immune-related functions.

### 3.5. Correlation between Risk Score Models and Somatic Cell Variants

**Figure 6:**
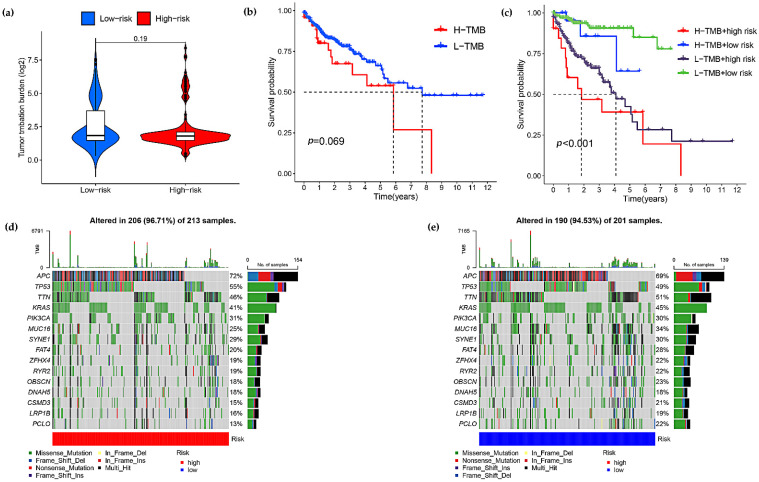
Correlations between the risk score model and somatic variants. (**a**) Differential analysis of tumor mutational burden (TMB) between high and low risk subgroups for colon adenocarcinoma. (**b**) Correlation between the prognosis and TMB in COAD patients. (**c**) Predictive power of risk model in high and low TMB groups. (**d**,**e**) Compare the mutation rates of reported prognosis-associated genes in low- and high-risk groups.

In view of the prognostic value of TMB, we tried to investigate the relationship between tumor mutational burden and risk score model, and the results showed no significant difference in TMB levels between high and low risk subgroups (Figure 6a), and also found no significant relationship between high and low TMB and survival of COAD patients (Figure 6b), next we evaluated the predictive ability of the risk model in high and low TMB groups. The results showed that the prognostic model had good predictive power in both the high and low TMB groups, indicating that the predictive model we constructed was not confounded by TMB status (Figure 6c).

In addition, we explored the mutation rates of prognosis-related genes in the high and low risk subgroups by waterfall plots, and APC, TP53 mutations were more frequent in the high-risk group, and TTN, KRAS, MUC16, FAT4, ZFHX4, RYR2, OBSCN, CSMD3, LRP1B, PCLO mutations were more frequent in the low-risk group (Figure 6d,e).

### 3.6. Screening of Potential Drugs for COAD

Further screening of potential drugs for COAD identified nine compounds that may be effective in COAD, namely ATRA, Axitinib, EHT1864, JW-7-24-1, OSI-930, Linifanib, PF-4708671, WZ3105, and Phenformin (Figure S1). The results of the bar graphs showed that the sensitivity of the nine compounds differed between the high- and low-risk groups, with the IC50 being significantly lower in all high-risk groups than in the low-risk group (*p* < 0.01). The correlation between risk scores and the sensitivity of these drugs was further investigated (Figure S2), and it was found that higher risk scores corresponded to lower IC50 values. These results suggest that patients in the high-risk group were more sensitive to the drugs, that patients in the high-risk group would benefit more from the potential drugs, and that there was also a negative correlation between the risk score and the sensitivity of the drugs.

It has been shown that down-regulation of sphingosine kinase 2 increases ATRA-induced RARβ expression, arrests the cell cycle in G1 phase, and induces apoptosis in colon cancer cells [35]. The combination of Axitinib and ABT263 exerted a synergistic effect on KRAS-mutant colon cancer cells by enhancing apoptosis [36]. Moreover, the combination of PF-4708671 and OSI-906 effectively inhibited the growth of OSI-906-resistant colon carcinoma Cells [37]. In conclusion, the above studies provide a basis for further research into potential drugs screened for the treatment of COAD.

### 3.7. Construction of ceRNA Network and PPI Network and Gene Set Enrichment Analysis

**Figure 7:**
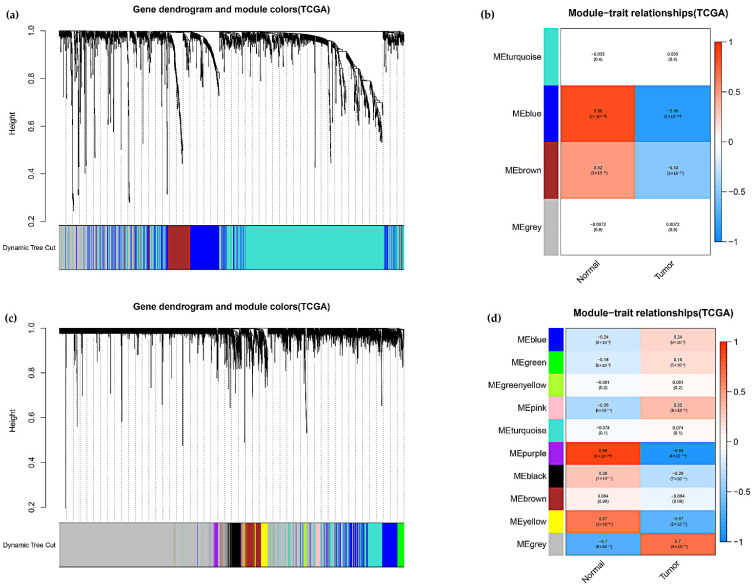
Screening for modules significantly associated with colon adenocarcinoma by WGCNA. (**a**) Hierarchical cluster analysis of the identified lncRNAs was performed to detect co-expression clusters with corresponding color assignments. (**b**) Correlation analysis of clinical features of colon adenocarcinoma with Eigengene of lncRNAs, where MEblue had the highest correlation coefficient. (**c**) Hierarchical clustering analysis of the identified mRNAs. (**d**) Correlation analysis of clinical features of colon adenocarcinoma with Eigengene of mRNAs, where MEpurple had the highest correlation coefficient.

**Figure 8:**
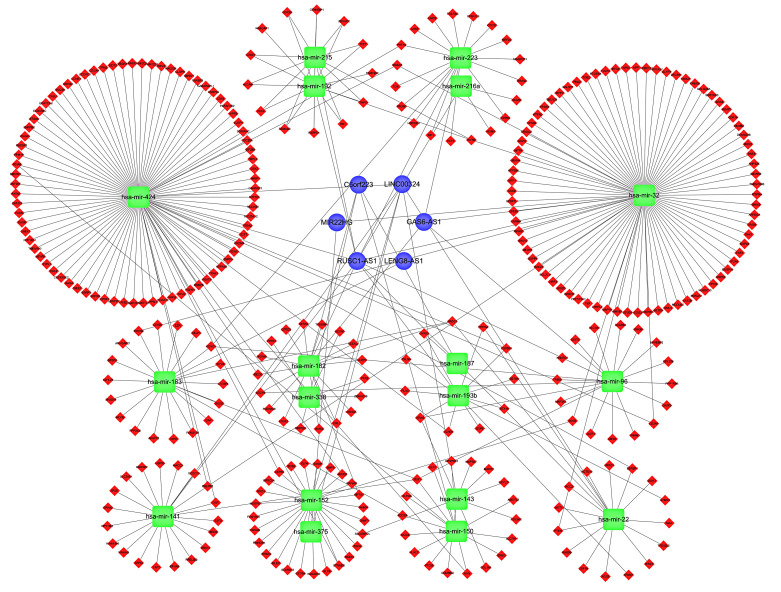
Construction of lncRNA–miRNA–mRNA ceRNA network.

**Figure 9:**
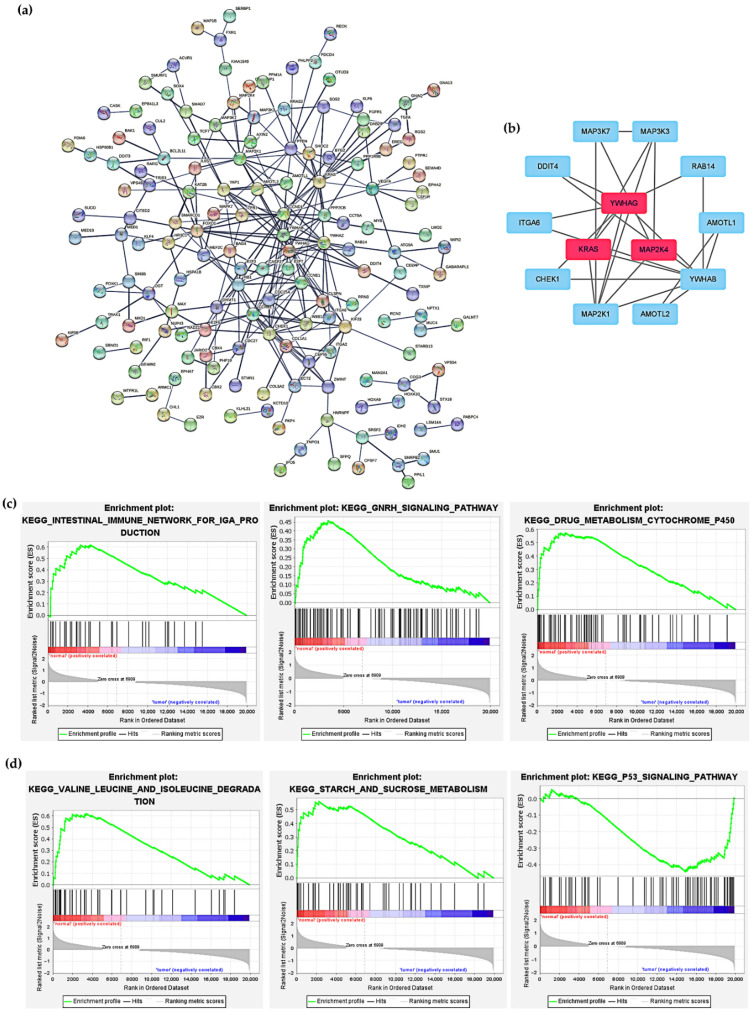
Construction of protein–protein interaction (PPI) network and gene set enrichment analysis. (**a**) PPI network was constructed with 325 targeting mRNAs. (**b**) PPI network of 13 genes were obtained by Cytoscape, among which YWHAG, KRAS, MAP2K4 were core genes. (**c**,**d**) Potential signaling pathways for targeting mRNAs revealed by GSEA analysis.

In colon adenocarcinoma, we further explored how cuproptosis-related lncRNAs regulate mRNA expression through miRNA targeting. First, the lncRNAs in the modules associated with the clinical features of colon adenocarcinoma were screened by WGCNA, and the results showed that Meblue had the highest correlation coefficient, so the Meblue module was selected (Figure 7a,b). Similarly, when probing the mRNAs of the related modules, MEpurple was also selected due to the highest correlation coefficient (Figure 7c,d). The 6 lncRNAs, 18 miRNAs and 325 mRNAs obtained from the screening were then used to construct a lncRNA-miRNA-mRNA ceRNA network (Figure 8). The 325 targeted mRNAs were used to construct a PPI network (Figure 9a), and the sub-network of PPI network was further constructed using the Mcode plugin in Cytoscape, from which three core genes YWHAG, KRAS, MAP2K4 were screened out (Figure 9b). To investigate the potential biological pathways involved in mRNA, we performed a gene set enrichment analysis (GSEA). *p* values < 0.05 were considered statistically significant. The results showed that the intestinal immune network pathway for iga production, the gnrh signaling pathway, the drug metabolism cytochrome p450, valine leucine and isoleucine degradation, as well as the starch and sucrose metabolism were active in the normal group. The p53 signaling pathway was active in the tumor group (Figure 9c,d). The above results provide us with new avenues to search for the potential functions of cuproptosis-related lncRNAs in colon adenocarcinoma.

## 4. Discussion

Cuproptosis is a regulated form of cell death that is distinct from other forms of cell death. It has been shown that genes associated with cuproptosis are favorable predictors of prognosis in related cancers [38]. However, lncRNAs have received less attention compared to genes involved in the regulation of cuproptosis. Study results show that lncRNAs play an important role in cancer development [15,39,40]. Considering the high heterogeneity among different types of colorectal cancer, coupled with the fact that colon adenocarcinoma is the predominant type, there is a need for a comprehensive assessment of the prognostic value of cuproptosis-related lncRNAs in colon adenocarcinoma. In addition, inconsistent treatment and prognosis at different stages of colon adenocarcinoma, as well as the influence of factors such as tumor mutation burden, interfere with the applicability of the prognostic model. Additionally, there is an urgent need for a predictive model that is highly accurate for different stages of colon adenocarcinoma.

In this study, we screened 15 lncRNAs closely associated with cuproptosis by various methods, including lasso regression analysis, and univariate and multivariate Cox analysis (Figure 1). Using these characteristic lncRNAs to construct a prognostic model of COAD (Figure 2), patients were divided into two subgroups of high and low risk, and the model was found to have high predictive accuracy by independent prognostic analysis and ROC curves. The model was further combined with independent prognostic factors (gender, age, and stage) to form a nomogram with better predictive power for overall survival. Meanwhile, the survival curve results showed that the model is applicable to multiple pathological features of COAD patients (Figure 3). Enrichment analysis identifies biological processes and pathways associated with immunity, leading to an in-depth comparison of immune-related functions between the high- and low-risk groups (Figure 4). Since TMB can be used as a prognostic predictor [41], we explored whether there were significant differences in TMB levels between the high-risk and low-risk groups and the implications for this model (Figure 5). Then, we constructed the lncRNA/miRNA/mRNA ceRNA network and PPI network to explore the potential biological mechanism of cuproptosis-related lncRNAs (Figure 6, Figure 7 and Figure 8).

In recent years, lncRNAs have received increasing attention. Several studies have found that lncRNAs play an important role in tumor progression, such as the down-regulation of linc01140, which inhibits proliferation and the invasion of osteosarcoma by targeting the miR-139-5p/HOXA9 axis [40]. Additionally, HOXA-AS2 is aberrantly expressed in malignant tumors such as gastric cancer, cholecystitis, hepatocellular carcinoma and breast cancer [42]. Among the 15 lncRNAs involved in this study, there were 10 unfavorable factors (AP003119.3, RNF216P1, AC156455.1, AL360270.1, AC139720. 2, AC092614.1, LRP4-AS1, AP003555.1, AL513550.1, AL512306.2) and 5 favorable factors (AC073896.3, LINC00511, AC026979.4, AC103703.1, LRP1-AS). In colon adenocarcinoma, AC073896.3 as a favorable factor [17], and AC156455.1 [14] and AP003555.1 [43] as unfavorable factors have been confirmed by other studies. Moreover, down-regulated LINC00511 obstructs the tumorigenesis of COAD through restoring miR-625-5p and silencing WEE1 [44]. LRP1-AS is associated with cancer development through the regulation of the pepsin receptor LRP1 [45].

Tumor mutational burden (TMB) represents the number of mutations per megabase (Mut/Mb) of DNA that were sequenced in a specific cancer. TMB has been shown to be a biomarker for immune checkpoint inhibitors (ICIs) in melanoma [46], and high TMB acts via anti-CTLA-4 and anti-PD-1 in melanoma and non-small cell lung cancer [41]. In cancers such as head and neck squamous cell carcinoma, prostate adenocarcinoma, bladder urothelial carcinoma and colorectal cancer, lower TMB has longer OS [47,48]. For example, among colorectal cancer patients treated with oxaliplatin chemotherapy and those treated with irinotecan chemotherapy, progression-free survival was significantly better in the low-TMB group (11.9 months vs. 6.5 months, *p* < 0.001), whereas there was no difference in PFS for high-TMB [49]. However, other studies have shown that high TMB is associated with better prognosis in colorectal cancer patients receiving adjuvant chemotherapy with fluoropyrimidine and oxaliplatin after curative surgery [50]. Despite the promising results, the predictive role of TMB is controversial due to the poor reproducibility of TMB results, the lack of routine testing in clinical practice, and the lack of extensive experimental validation support [51]. It is worth mentioning that, in the study, we also found no difference in TMB between the high-risk and low-risk groups, and the degree of TMB did not interfere with the prediction of the high-risk and low-risk groups. The above results show that the constructed model was not confounded by TMB status.

This study established a new prognostic model of cuproptosis-related lncRNA, which provides some reference for the treatment of COAD patients, but there are still some problems. Firstly, the constructed prognostic model, despite having high accuracy, lacked data support for further in vivo and in vitro experiments. Finally, the intrinsic mechanism of how these lncRNAs affect cuproptosis is still unknown.

## 5. Conclusions

This prognostic model constructed based on 15 cuproptosis-related lncRNAs is a reliable method for predicting clinical outcomes in patients with colon adenocarcinoma. Additionally, our study helps to explore the potential mechanism of cuproptosis-related lncRNA interaction with colon adenocarcinoma. It deserves further study.

## Figures and Tables

**Table 1:** 15 prognosis-related lncRNAs obtained based on multivariate Cox regression analysis.

lncRNA Name	Coefficient	HR	HR.95L	HR.95H	*p* Value
AP003119.3	2.443247512	4.291960	1.570556	11.728919	0.004510
RNF216P1	1.367425711	3.088040	1.200965	7.940272	0.019283
AC156455.1	0.733807815	1.681309	1.150079	2.457917	0.007326
AL360270.1	1.773933174	2.986836	1.163762	7.665821	0.022887
AC073896.3	−2.343500132	0.356807	0.151339	0.841232	0.018520
AC139720.2	1.047691955	1.814512	1.159697	2.839062	0.009090
AC092614.1	1.213541229	2.343952	1.077011	5.101259	0.031797
LINC00511	−1.445411714	0.150115	0.062617	0.359881	0.000021
LRP4-AS1	1.80630443	1.799438	1.119335	2.892770	0.015292
AC026979.4	−3.402432218	0.029192	0.001764	0.483129	0.013586
AC103703.1	−4.616328154	0.016495	0.000326	0.833521	0.040272
AP003555.1	0.58763672	2.636331	1.595781	4.355387	0.000154
AL513550.1	1.525410885	2.399151	1.194502	4.818681	0.013914
LRP1-AS	−7.45422088	0.083526	0.007523	0.927419	0.043247
AL512306.2	2.464575716	4.895259	1.643549	14.580379	0.004341

**Table 2:** Specific baseline clinical characteristic of 446 COAD patients.

Covariates	Type	446 Patients	Validation Group	Training Group	*p* Value
Age	<60 years	121	56	65	0.1236
	≥60 years	325	167	158	
gender	FEMALE	212	101	111	0.3935
	MALE	234	122	112	
stage	Stage I	75	35	40	0.6808
	Stage II	175	93	82	
	Stage III	124	64	60	
	Stage IV	61	28	33	
	unknown	11	3	8	
Pathologic T stage	T1	10	3	7	0.6372
	T2	76	38	38	
	T3	303	153	150	
	T4	56	29	27	
	unknown	1	0	1	
Pathologic M stage	M0	329	169	160	0.519
	M1	61	28	33	
	unknown	56	26	30	
Pathologic N stage	N0	265	135	130	0.8764
	N1	102	49	53	
	N2	79	39	40	

## Data Availability

The data presented in this study can be found in the TCGA database (https://portal.gdc.cancer.gov, accessed on 23 April 2022).

## Supplementary Materials

The following supporting information can be downloaded at: https://www.mdpi.com/article/10.3390/curroncol29090517/s1, Figure S1: Analysis of chemotherapy response (a–c). Boxplots of the difference in half-inhibitory concentration (IC50) values of potential drugs screened between high and low risk groups. Figure S2: Correlation analysis between risk score and drug sensitivity (a–c). The potential compounds were negatively correlated with the risk score. The higher the risk score, the higher the drug sensitivity (x-axis is the risk score and y-axis is the result of drug sensitivity. Spearman analysis was used). File S1: Expression files of cuproptosis-related genes. File S2: Expression file of cuproptosis-related lncRNAs. File S3: File of combined cuproptosis-related lncRNAs and clinical data.

## References

[1] Sung H., Ferlay J., Siegel R.L. (2021). Global Cancer Statistics 2020: GLOBOCAN Estimates of Incidence and Mortality Worldwide for 36 Cancers in 185 Countries. CA A Cancer J. Clin..

[2] Siegel R.L., Miller K.D. (2021). Cancer Statistics, 2021. CA Cancer J. Clin..

[3] Barresi V., Bonetti L.R., Ieni A., Caruso R.A., Tuccari G. (2015). Histological grading in colorectal cancer: New insights and perspectives. Histol. Histopathol..

[4] Pita-Fernández S., González-Sáez L., López-Calviño B., Seoane-Pillado T., Rodríguez-Camacho E., Pazos-Sierra A., González-Santamaría P., Pértega-Díaz S. (2016). Effect of diagnostic delay on survival in patients with colorectal cancer: A retrospective cohort study. BMC Cancer.

[5] Voli F., Valli E. (2020). Intratumoral Copper Modulates PD-L1 Expression and Influences Tumor Immune Evasion. Cancer Res..

[6] Zhang X., Yang Q. (2018). Association between serum copper levels and lung cancer risk: A meta-analysis. J. Int. Med. Res..

[7] Pavithra V., Sathisha T.G., Kasturi K., Mallika D.S., Amos S.J., Ragunatha S. (2015). Serum levels of metal ions in female patients with breast cancer. J. Clin. Diagn. Res. JCDR.

[8] Baltaci A.K., Dundar T.K., Aksoy F., Mogulkoc R. (2017). Changes in the Serum Levels of Trace Elements Before and After the Operation in Thyroid Cancer Patients. Biol. Trace Elem. Res..

[9] Shanbhag V.C., Gudekar N., Jasmer K., Papageorgiou C., Singh K., Petris M.J. (2021). Copper metabolism as a unique vulnerability in cancer. Biochim. Biophys. Acta Mol. Cell Res..

[10] Tsvetkov P., Coy S. (2022). Copper induces cell death by targeting lipoylated TCA cycle proteins. Science.

[11] Ulitsky I., Bartel D.P. (2013). lincRNAs: Genomics, evolution, and mechanisms. Cell.

[12] Alexander R.P., Fang G., Rozowsky J., Snyder M., Gerstein M.B. (2010). Annotating non-coding regions of the genome. Nat. Rev. Genet..

[13] Peng W.X., Koirala P., Mo Y.Y. (2017). LncRNA-mediated regulation of cell signaling in cancer. Oncogene.

[14] Chai X.K., Qi W., Zou C.Y., He C.X., Su M., Zhao D.Q. (2021). Potential Prognostic Value of a Seven m6A-Related LncRNAs Signature and the Correlative Immune Infiltration in Colon Adenocarcinoma. Front. Genet..

[15] Li J., Xiang R., Song W., Wu J., Kong C., Fu T. (2022). A Novel Ferroptosis-Related LncRNA Pair Prognostic Signature Predicts Immune Landscapes and Treatment Responses for Gastric Cancer Patients. Front. Genet..

[16] Liu L., Huang L., Chen W., Zhang G., Li Y., Wu Y., Xiong J., Jie Z. (2022). Comprehensive Analysis of Necroptosis-Related Long Noncoding RNA Immune Infiltration and Prediction of Prognosis in Patients With Colon Cancer. Front. Mol. Biosci..

[17] Zhou W., Zhang S. (2020). Development of Prognostic Indicator Based on Autophagy-Related lncRNA Analysis in Colon Adenocarcinoma. BioMed Res. Int..

[18] Sun J., Li L., Chen H., Gan L., Guo X., Sun J. (2022). Identification and Validation of an m7G-Related lncRNAs Signature for Prognostic Prediction and Immune Function Analysis in Endometrial Cancer. Genes.

[19] Meier L., Van De Geer S., Bühlmann P. (2008). The group lasso for logistic regression. J. R. Stat. Soc. Ser. B (Stat. Methodol.).

[20] Long J., Zhang L., Wan X., Lin J., Bai Y., Xu W., Xiong J., Zhao H. (2018). A four-gene-based prognostic model predicts overall survival in patients with hepatocellular carcinoma. J. Cell. Mol. Med..

[21] Li H., Gao C., Liu L., Zhuang J., Yang J., Liu C., Zhou C., Feng F., Sun C. (2019). 7-lncRNA Assessment Model for Monitoring and Prognosis of Breast Cancer Patients: Based on Cox Regression and Co-expression Analysis. Front. Oncol..

[22] Park S.Y. (2018). Nomogram: An analogue tool to deliver digital knowledge. J. Thorac. Cardiovasc. Surg..

[23] Harrell F.E., Lee K.L., Mark D.B. (1996). Multivariable prognostic models: Issues in developing models, evaluating assumptions and adequacy, and measuring and reducing errors. Stat. Med..

[24] Zhi S., Yang B., Zhou S., Tan J., Zhong G., Han F. (2022). Immune-Related LncRNAs to Construct a Prognosis Risk-Assessment Model for Gastric Cancer. Curr. Oncol..

[25] Xiao B., Liu L., Li A., Xiang C., Wang P., Li H., Xiao T. (2020). Identification and Verification of Immune-Related Gene Prognostic Signature Based on ssGSEA for Osteosarcoma. Front. Oncol..

[26] Geeleher P., Cox N., Huang R.S. (2014). pRRophetic: An R package for prediction of clinical chemotherapeutic response from tumor gene expression levels. PLoS ONE.

[27] Geeleher P., Cox N.J., Huang R.S. (2014). Clinical drug response can be predicted using baseline gene expression levels and in vitro drug sensitivity in cell lines. Genome Biol..

[28] Jeggari A., Marks D.S., Larsson E. (2012). miRcode: A map of putative microRNA target sites in the long non-coding transcriptome. Bioinformatics.

[29] von Mering C., Huynen M., Jaeggi D., Schmidt S., Bork P., Snel B. (2003). STRING: A database of predicted functional associations between proteins. Nucleic Acids Res..

[30] Ahmadi M., Pashangzadeh S., Mousavi P., Saffarzadeh N., Habibi M.A., Hajiesmaeili F., Rezaei N. (2021). ACE2 correlates with immune infiltrates in colon adenocarcinoma: Implication for COVID-19. Int. Immunopharmacol..

[31] Wilson B.G., Roberts C.W. (2011). SWI/SNF nucleosome remodellers and cancer. Nat. Rev. Cancer.

[32] Chen C., Enomoto A. (2020). Complex roles of the actin-binding protein Girdin/GIV in DNA damage-induced apoptosis of cancer cells. Cancer Sci..

[33] Qin L., Chen C., Chen L., Xue R., Ou-Yang M., Zhou C., Zhao S., He Z., Xia Y., He J. (2017). Worldwide malaria incidence and cancer mortality are inversely associated. Infect. Agents Cancer.

[34] Chen L., He Z., Qin L., Li Q., Shi X., Zhao S., Chen L., Zhong N., Chen X. (2011). Antitumor effect of malaria parasite infection in a murine Lewis lung cancer model through induction of innate and adaptive immunity. PLoS ONE.

[35] Chu J.H., Gao Z.H., Qu X.J. (2014). Down-regulation of sphingosine kinase 2 (SphK2) increases the effects of all-trans-retinoic acid (ATRA) on colon cancer cells. Biomed. Pharmacother..

[36] Wang G., Huang Y., Wu Z., Zhao C., Cong H., Ju S., Wang X. (2019). KRAS-mutant colon cancer cells respond to combined treatment of ABT263 and axitinib. Biosci. Rep..

[37] Zhang Y., Wang Q., Chen L., Yang H.S. (2015). Inhibition of p70S6K1 Activation by Pdcd4 Overcomes the Resistance to an IGF-1R/IR Inhibitor in Colon Carcinoma Cells. Mol. Cancer Ther..

[38] Bian Z., Fan R. (2022). A Novel Cuproptosis-Related Prognostic Gene Signature and Validation of Differential Expression in Clear Cell Renal Cell Carcinoma. Genes.

[39] Li H., Liu L., Huang T., Jin M., Zheng Z., Zhang H., Ye M., Liu K. (2021). Establishment of a novel ferroptosis-related lncRNA pair prognostic model in colon adenocarcinoma. Aging.

[40] Zhang S., Chen R. (2022). LINC01140 regulates osteosarcoma proliferation and invasion by targeting the miR-139-5p/HOXA9 axis. Biochem. Biophys. Rep..

[41] Chan T.A., Yarchoan M., Jaffee E., Swanton C., Quezada S.A., Stenzinger A., Peters S. (2019). Development of tumor mutation burden as an immunotherapy biomarker: Utility for the oncology clinic. Ann. Oncol. Off. J. Eur. Soc. Med. Oncol..

[42] Wang J., Su Z., Lu S., Fu W., Liu Z., Jiang X., Tai S. (2018). LncRNA HOXA-AS2 and its molecular mechanisms in human cancer. Clin. Chim. Acta Int. J. Clin. Chem..

[43] Wu Z., Lu Z., Li L., Ma M., Long F., Wu R., Huang L., Chou J., Yang K., Zhang Y. (2021). Identification and Validation of Ferroptosis-Related LncRNA Signatures as a Novel Prognostic Model for Colon Cancer. Front. Immunol..

[44] Qian X., Jiang C., Zhu Z., Han G., Xu N., Ye J., Wang R. (2022). Long non-coding RNA LINC00511 facilitates colon cancer development through regulating microRNA-625-5p to target WEE1. Cell Death Discov..

[45] Samuels T.L., Zimmermann M.T., Zeighami A., Demos W., Southwood J.E., Blumin J.H., Bock J.M. (2021). RNA Sequencing Reveals Cancer-Associated Changes in Laryngeal Cells Exposed to Non-Acid Pepsin. Laryngoscope.

[46] Addeo A., Friedlaender A., Banna G.L., Weiss G.J. (2021). TMB or not TMB as a biomarker: That is the question. Crit. Rev. Oncol./Hematol..

[47] Liu L., Bai X., Wang J., Tang X.R., Wu D.H., Du S.S., Du X.J., Zhang Y.W., Zhu H.B., Fang Y. (2019). Combination of TMB and CNA Stratifies Prognostic and Predictive Responses to Immunotherapy Across Metastatic Cancer. Clin. Cancer Res. Off. J. Am. Assoc. Cancer Res..

[48] Lv J., Zhu Y., Ji A., Zhang Q., Liao G. (2020). Mining TCGA database for tumor mutation burden and their clinical significance in bladder cancer. Biosci. Rep..

[49] Pai S.G., Carneiro B.A., Chae Y.K., Costa R.L., Kalyan A., Shah H.A., Helenowski I., Rademaker A.W., Mahalingam D., Giles F.J. (2017). Correlation of tumor mutational burden and treatment outcomes in patients with colorectal cancer. J. Gastrointest. Oncol..

[50] Lee D.W., Han S.W. (2019). Tumor Mutation Burden and Prognosis in Patients with Colorectal Cancer Treated with Adjuvant Fluoropyrimidine and Oxaliplatin. Clin. Cancer Res..

[51] Bravaccini S., Bronte G. (2021). TMB in NSCLC: A Broken Dream?. Int. J. Mol. Sci..

