# Supplementary material for: In Silico Identification and Validation of Cuproptosis-Related LncRNA Signature as a Novel Prognostic Model and Immune Function Analysis in Colon Adenocarcinoma
Source: Curr Oncol. 2022 Sep 15;29(9):6573–93. doi: 10.3390/curroncol29090517 (PMC9497598; doi:10.3390/curroncol29090517)
Supplement: Supplementary file 1 [file curroncol-29-00517-s001.zip › curroncol-1869125-supplementary figures.pdf]

Current Oncology

MDPI

Article

In Silico Identification and Validation of Cuproptosis-Related LncRNA Signature as a Novel Prognostic Model and Immune Function Analysis in Colon Adenocarcinoma

Yue Wang, Xulong Huang, Siyu Chen, Huajuan Jiang, Huanan Rao, Lijie Lu, Feiyan Wen and Jin Pei

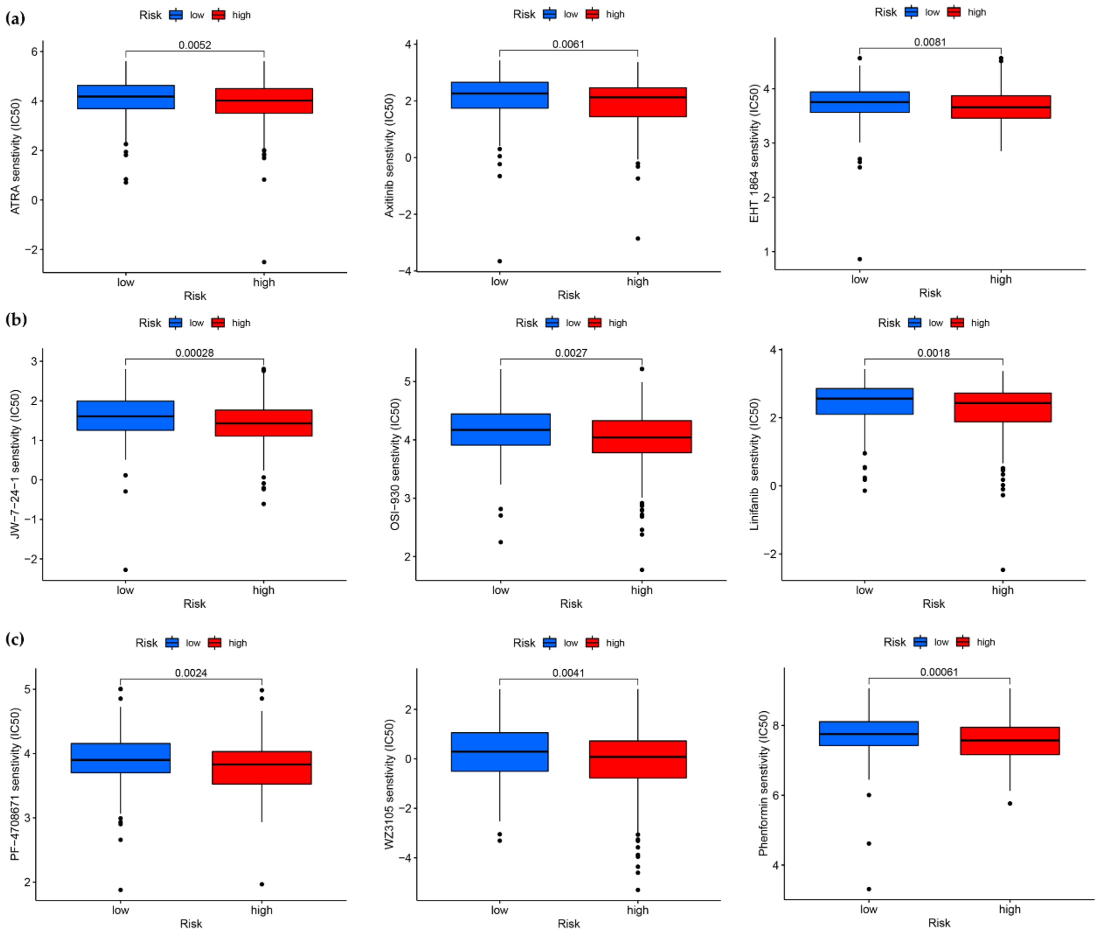

Figure S1. Analysis of chemotherapy response (a-c) Boxplots of the difference in half-inhibitory concentration (IC50) values of potential drugs screened between high and low risk groups.

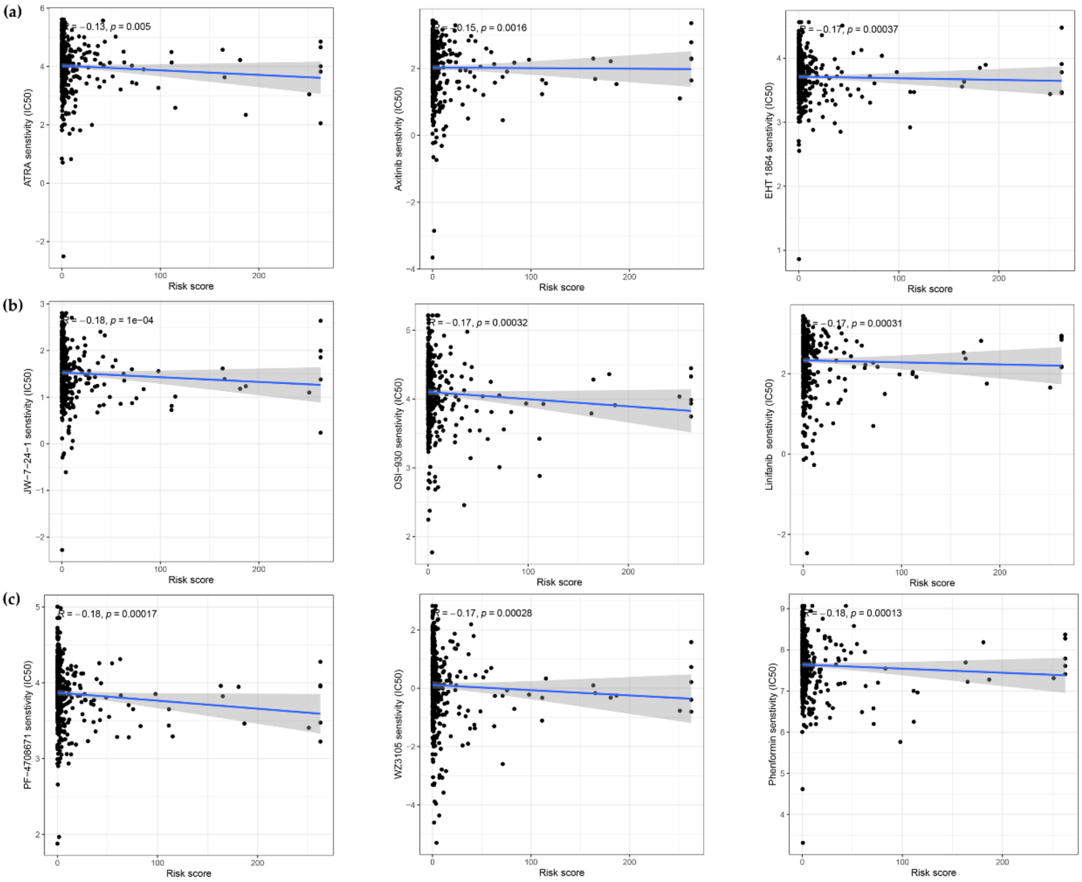

Figure S2. Correlation analysis between risk score and drug sensitivity (a-c) The potential compounds were negatively correlated with the risk score. The higher the risk score, the higher the drug sensitivity. (x-axis is the risk score and y-axis is the result of drug sensitivity. Spearman analysis was used).
